# Are Sexist Attitudes and Gender Stereotypes Linked? A Critical Feminist Approach With a Spanish Sample

**DOI:** 10.3389/fpsyg.2019.02410

**Published:** 2019-10-24

**Authors:** Rubén García-Sánchez, Carmen Almendros, Begoña Aramayona, María Jesús Martín, María Soria-Oliver, Jorge S. López, José Manuel Martínez

**Affiliations:** ^1^Department of Social Psychology and Methodology, Autonomous University of Madrid, Madrid, Spain; ^2^Department of Biological and Health Psychology, Autonomous University of Madrid, Madrid, Spain; ^3^Faculty of Health Sciences, International University of La Rioja, Logroño, Spain; ^4^Department of Psychology and Pedagogy, Public University of Navarre, Pamplona, Spain

**Keywords:** sexism, gender stereotype, psychometric properties, invariance, Spanish men and women

## Abstract

This study aims to verify the psychometric properties of the Spanish versions of the Social Roles Questionnaire (SRQ; Baber and Tucker, 2006), Modern Sexism Scale (MS), and Old-Fashioned Sexism Scale (OFS; Swim et al., 1995; Swim and Cohen, 1997). Enough support was found to maintain the original factor structure of all instruments in their Spanish version. Differences between men and women in the scores are commented on, mainly because certain sexist attitudes have been overcome with greater success in the current Spanish society, while other issues, such as distribution of power in organizational hierarchies or distribution of tasks in the household, where traditional unequal positions are still maintained. In all cases, it was found that men showed greater support for sexist attitudes. The correlations between the three instruments were as expected in assessing sexist attitudes that tend to relate to each other. Eventually, we found no empirical evidence for the postulated link between sexist attitudes and traditional gender stereotypes. Our results call for the validity and effectiveness of the classic theories of gender psychology, such as gender schema theories (Bem, 1981; Markus et al., 1982) and the notion of a gender belief system (Deaux and Kite, 1987; Kite, 2001).

## Introduction

The American Psychological Association (2011, 2015) recommends examining gender differences; however, it does not include any guide about how to interpret these differences in empirical studies. This situation results in the confusion of the terms “sex” and “gender” in academic and scientific texts (Pryzgoda and Chrisler, 2000; Cowan, 2005; Wickes and Emmison, 2007; Hammarstrom and Annandale, 2012). Westbrook and Saperstein (2015) have shown the lack of sensitivity in the recognition of sexual and gender diversity in Social Sciences investigations, which not only results in a lack of recognition of persons not adjusting to the binarism of the sex/gender/sexuality system (Butler, 1990, 2004), but also in a serious bias in the production of scientific knowledge (Balarajan et al., 2011). To counteract these deficiencies Hesse-Biber and Leavy (2008) described some methodological approaches that can be very helpful when performing the design of a research from a feminist epistemological perspective. There are also several statistical considerations to be made to improve the analysis of the data obtained in an empirical study: from the inclusion of sex as a moderator variable in the regression analyses (e.g., Baron and Kenny, 1986; Orue et al., 2016), to the application of structural equation models (e.g., factorial invariance) which allow a more complex, sophisticated analysis of gender differences (Byrne, 2008).

In line with a critical feminist approach, our aim was the assessment and the analysis of the relation between the attitudes on gender roles and gender stereotypes in a Spanish sample of women and men. We have taken as a frame of analysis the Social Role Theory (Eagly, 1987), the Gender Schema Theories (Bem, 1981; Markus et al., 1982; Spence, 1985), and the Gender Belief System (Deaux and Kite, 1987; Kite, 2001). We have chosen this theoretical framework as they are the main proposals in Gender Psychology that have established some type of relationship between the social changes in women’s position in society, the adoption of traditional gender roles and stereotypes and maintaining sexist attitudes about men and women. By such, we intended to contribute to the study of gender in Spain, responsibly using the most appropriate procedures for the analysis of differences between men and women on the basis of the results of instruments of assessment of sexist attitudes and gender stereotypes.

### Exploring the Relation Between Gender Role Attitudes and Gender Stereotypes

#### Social Role Theory

The Social Role Theory is based on the assumption that in every community tasks are divided assigning different roles and responsibilities according to the sex/gender of the persons (Eagly and Wood, 1991, 1999; Eagly et al., 2000; Wood and Eagly, 2002). This labor division would become the backbone for the social structure of the community (Wood and Eagly, 2010, 2012), thus generating social inequalities according to the privileges, rights, and obligations associated with each sex/gender. Therefore, gender stereotypes would be given content and would define the expectations about the appropriate behaviors, traits or attitudes for men and women (Eagly et al., 2000). Based on these gender roles, gender identity would emerge (Wood and Eagly, 2009).

In Western societies, a patriarchal system predominates where roles associated with men are linked to a higher recognition and status, and roles associated with women are worse rated (Ridgeway and Bourg, 2004; Guimond, 2008). Political and legislative changes should produce significant changes in gender roles (Eagly and Wood, 1991, 1999; Eagly et al., 2000; Wood and Eagly, 2002) and these changes should also induce modification in the attitudes toward these gender roles (Eagly and Chaiken, 2007).

#### Gender Schema Theories

The Gender Schema Theory by Sandra Bem (1981) proposes that gender identity stems from the scheme the individual has about the roles assigned to men and women. These schemes are the stereotypes which organize the knowledge about men and women, including physical characteristics and personality traits associated with men and women prototype, respectively.

According to Bem’s proposal, persons identifying with their traditional gender role (women with feminine characteristics and male with masculine characteristics) tend to organize the information in dichotomous masculine-feminine terms. Later, Markus et al. (1982) made a change in this theory, highlighting that, irrespective of their biological sex, persons with masculine characteristics would process the information associated with the masculine stereotype from their own scheme, feminine persons would use their scheme with the information associated with the feminine condition, androgynous persons would do it with both types of information and “undifferentiated” persons would not process any information schematically.

However, the review of the investigations that have analyzed the scores in femininity and masculinity scales as predictors for behavior has not sufficiently supported these theories (i.e., Deaux et al., 1985; Frable and Bem, 1985; Beauvais and Spence, 1987; Payne et al., 1987). This led Janet Spence (1985) to propose an alternative model postulating a multidimensional approach to the study of masculinity and femininity. This model involved the creation of new instruments considering the different dimensions of the constructs (that is, Attitudes Toward Women Scale, Spence and Helmreich, 1972; Male-Female Relations Questionnaire, Spence et al., 1980). From this perspective, the Bem Sex Role Inventory (BSRI) and Personnel Attributes Questionnaire (PAQ) instruments would be defined as self-description measures in terms of “communal” and “agentic” traits. The main postulate of Spence was the consideration of gender as a multifactorial construct comprising attitudes, traits, interests, preferences and behaviors associated with men and women in society. However, the relationship between these elements does not need to be univocal and solid, but depends on other factors that may affect their transformation.

#### Gender Belief System

Based on the gender belief system model (Deaux and Kite, 1987; Kite, 2001) it is proposed that our views about men and women are conditioned by social expectations. The belief system includes the gender stereotypes, the attitudes toward the roles appropriate for each gender and the views about persons breaching these expectations. The concept of masculinity and femininity is bipolar, so that someone described with stereotyped masculine traits is also expected to have masculine physical characteristics and to adopt a masculine gender role (Deaux and Lewis, 1984; Berndt and Heller, 1986). Therefore, roles, traits and appearance form a consistent, interrelated system: it is associated with men having traits related to competence (i.e., confidence, independence or control) and women holding traits related to emotional expressiveness (i.e., warmness, kindness or concern for others) (Spence and Helmreich, 1978; Williams and Best, 1990); with regard to physical characteristics, men are expected to be stronger and have broader shoulders, and women more gentle and elegant; in turn, over the gender roles assigned, men are responsible for economic aspects and for making decisions, while women are assigned household tasks and care for others (Deaux and Lewis, 1984).

### Assessing Sexist Attitudes and Gender Stereotypes

The study of gender stereotypes and the attitudes about gender roles is an area significantly developed in recent decades. For the most part, to be noted is the use of self-reports as the preferred assessment method in the studies intending to approach gender stereotypes and attitudes about gender roles (Smiler and Epstein, 2010). There are a number of measures that have been used. The most widely used are described herein.

#### Internalization of Stereotypes

Bem Sex Role Inventory (Bem, 1974). Together with the PAQ (Spence et al., 1975), it is the most commonly used instrument for the assessment of gender stereotypes (Smiler and Epstein, 2010). The stated initial purpose of Sandra Bem was, in line with the US feminist movement of the 70s, to promote a more liberal view of sexuality noting that both men and women could have characteristics of masculinity and femininity, thus resulting in the concept of androgynous personality (Bem, 1972). In its origin, the BSRI included 60 personality traits: 20 characteristics associated with the feminine gender stereotype (e.g., “compassionate,” “tender”), other 20 which make reference to the masculine gender stereotype (e.g., “assertive,” “strong”) and the last 20 considered as neutral to both gender stereotypes (e.g., “conventional,” “adaptable”). When the feminine scale score is significantly higher than the masculine score, the person is defined as “feminine,” and vice versa. When there are no significant differences but in both scales the score is above the median, the person is classified as “androgynous,” while if both scores are below the median, it is called “undifferentiated.” For the most part, studies have found that the “masculinity” and “femininity” scales are not significantly correlated (Aguíñiga et al., 1987; Lenney, 1991), and that men and women score significantly higher in the scales consistent with their sex (Lenney, 1991). Hoffman and Borders (2001) performed a critical review of the last 25 years of use of the BSRI and concluded that the sociohistorical context in Western countries has considerably changed in recent years in respect to what was considered feminine and masculine at the time when the questionnaire was designed. However, the list of items has not been updated or reformulated. Furthermore, many authors have used the BSRI for the purpose of measuring masculinity and femininity, when it has been already extensively demonstrated that masculinity and femininity are broader concepts than the degree of adjustment to masculine roles (instrumental roles) and feminine roles (expressive traits) (e.g., Spence, 1985). Considering that factorial analyses have shown inconsistent results (Choi and Fuqua, 2003), together with the possible obsolescence of the items, caution is recommended when interpreting the results derived from its use (Smiler and Epstein, 2010).

#### Gender Role Attitudes

Old-Fashioned Sexism (OFS) and Modern Sexism Scales (MS) (Swim et al., 1995; Swim and Cohen, 1997). Both instruments arose from a previous study on racist attitudes. The OFS was designed to evaluate the most evident forms of sexism and the MS for detecting the most subtle aspects, such as resentment toward policies and practices seeking to tackle inequalities between men and women in the society. The participants are asked about their degree of agreement with a number of sentences that represent sexist attitudes about men and women. Good psychometric indicators have been reported in their extensive use (Smiler and Epstein, 2010).

Ambivalent Sexism Inventory (ASI; Glick and Fiske, 1996). It was used to evaluate hostile and benevolent sexism. The authors defined hostile sexism as a prejudice that places women as adversaries to men. Benevolent sexism would be defined as a manifestation through which men would protect women due to their presumed incompetence outside the area of intimacy and care for others. There are items about protective paternalism, complementary gender differences and heterosexual intimacy.

Social Roles Questionnaire (SRQ; Baber and Tucker, 2006). Due to its more recent publication, this questionnaire still shows a reduced use in empirical studies. However, its proposal intends to be an advance that can enrich the study of gender roles and sexist attitudes. With this regard, the authors point to flaws in previous instruments, i.e., being obsolete and reproducing a dichotomous gender view. Based on a social constructivist perspective, they proposed this new instrument for the assessment of attitudes on the social roles in the United States society, intending to overcome these limitations. This instrument included references to behaviors associated with men or women, as well as other items intending to gather more subtle or hidden attitudes to support gender inequality. In its original version, the instrument included three subscales: “General,” “Childhood,” and “Gender Transcendent” (GT) The first two were subsequently joined obtaining a final two-factor proposal.

### Current Social Position of Women in Spain

The feminist movement in Spain has forced to the political parties to achieve a commitment with the gender equality. However, the real equality still requires a major effort, as the current situation in Spain is not fair and equal for women. According to the most current data provided by the Spanish Instituto de la Mujer (2016), we provide a list of the indicators we consider most relevant to reflect the position of women in Spanish society today.

#### Educational System

The percentage of women enrolled in Primary and Secondary Education has remained stable, around 48%, in the past 20 years. However, the number of women enrolled in high school has decreased progressively to two percentage points from the academic year 1998–1999 (54.5%) to the academic year 2013–2014 (52.4%).

According to the different educational routes in the Spanish educational system, Intermediate Level and Advanced Level Vocational Training courses are offered, with different specialties. In both educational routes, it is seen that the branches where women are more represented were “Personal Image” with about 94% of women enrolled, “Social Services to the Community” with about 86% and “Textiles, Clothing Production and Leather” with about 88% women enrolled. This distribution in the different specialties has remained stable in the past 15 years.

According to the percentage of women enrolled in university degrees, we find 4.38% over the total enrollments in the academic year 2013–2014, which has remained stable in the past 10 years. According to the field of education, from higher to lower, in “Health Sciences” 69.6% are women, in “Arts and Humanities” 61.5%, in “Social and Legal Sciences” we find 60.4%, in “Sciences” 51.6% and in “Engineering and Architecture” 26.1%. Again, this distribution has remained unchanged in the past 10 years.

#### Labor Market

According to the last data for 2016, the fields of activity with a higher percentage of women are “Activities of households such as employers of domestic workers” (88.9%), “Human Health and Social Work Activities” (77.1%), and “Education” (66.6%). The fields with the lowest presence of women are “Building” (7%), “Extraction Industries” (10.5%), and “Water supplies and sanitation activities” (16.3%). In all cases, very similar percentages have persisted in the past 10 years.

By type of working day, part-time jobs have been still dominated by women in the past year. In 2015, 72.5% of the population working part time were women, vs. 40.3% of women working full time. Since 2005, a minor variation has taken place in this distribution, as then there were 78.1% women working part time and 34.6% working full time.

The type of contract has also changed slightly since 2005. Then 40.3% of the permanent contracts and 44.7% of the temporary contracts were signed by women. In 2015, however, both figures increased, 47.8% of women with permanent contracts and 48% of women with temporary contracts.

With regard to salaries, since the year 2004 a negative difference persists, around 18.8%, when comparing the hourly earnings per hour of women vs. men. The average pay gap in the European Union is 16.1%.

#### Reconciliation of Work With Family Life

The percentage of women requesting leaves of absence for caring for their relatives has remained unchanged since 2005, around 84% vs. men. With regard to leaves of absence for the care of children, the situation is similar, with 96.7% of women in 2005 and 93.3% in 2015.

According to the Survey on use of time (2009–2010), women dedicate 4 h 7′ to “home and family” vs. 1 h 54′ dedicated by men. The tasks to which women devote more time are: 1 h 24′ to “cooking activities,” 49′ to “home maintenance” and 32′ to “children care”; while men devote a longer time to “cooking activities” (26′) and “purchases and services” (17′).

#### Leading Positions

We can analyse the presence of women in state agencies and large companies to reflect the access level of women to leading positions.

• Women in senior positions of the Central State Administration have turned from 14.4% in 1995 to 32% in 2014.
• Women in IBEX-35 companies boards of directors have turned from 2.6% in 2004 to 18.2% in 2014.
• In the justice system, counting the positions of public prosecutors, secretaries of the courts and members of the judiciary, women represented 41.3% in 1995 and 58.8% in 2014.
• The percentage of women in boards of directors in Royal Academies is still much lower than in men; in 2015, we find 10.6% of women, that was 9.3% in 2011.
• The percentage of women in the Spanish Parliament has changed markedly, from 5.1% in the 1979–1982 term of office to 35.7% in 2011–2015.

### This Study

We have used three instruments of assessment of sexist attitudes that were translated into Spanish: SRQ (Baber and Tucker, 2006), OFS, and MS (Swim et al., 1995; Swim and Cohen, 1997). We have chosen these instruments for various reasons: the SRQ is one of the most current approaches to the study of sexist attitudes and adequate psychometric properties have been reported in a similar population in the United States; on the other hand, the OFS and the MS, even being older, are two very accurate instruments in the study of sexist attitudes in their two modalities and have been used in multiple studies (Smiler and Epstein, 2010) thus becoming two of the most important instruments in the field.

First, through confirmatory factor analysis (CFA), we intended to determine whether the Spanish versions of the scales had an internal structure similar to the original versions. We expected to confirm the two-factor structure for the SRQ and the one-factor models for both the OFS and MS scales, as originally proposed and further supported in the literature.

Second, we intended to examine the differences between men and women in their mean scores in the items of the SRQ, OFS, and MS. Consistent with prior findings (Swim et al., 1995; Swim and Cohen, 1997; Baber and Tucker, 2006), we expected men to have higher scores than women, thus showing greater support to sexist attitudes. We explored the equivalence between men and women in the factorial structures of the SRQ, OFS, and MS.

Finally, we intended to determine the relationships among the three instruments scores and whether upholding traditional gender stereotypes was empirically related to the SRQ, OFS, and MS scales scores. The internalization of the gender stereotypes was evaluated by the BSRI (Bem, 1974). In this respect: (1) We expected high positive correlations between SRQ, OFS, and MS in both men and women, considering that, independently, both SRQ (Baber and Tucker, 2006) and MS and OFS (Swim et al., 1995) have shown significant relationships with other instruments that evaluate sexist attitudes, such as AWS and ASI (Ogletree, 2015); (2) We classified the sample according to gender stereotypes evaluated by the BSRI and we wish to confirm the following hypothesis: (a) According to the Gender Social Role Theory (Eagly, 1987) we could expect that both men and women show the same support level to the different sexist attitudes evaluated with the instruments used. Despite the social achievements reached in matters of gender equality, there are several elements (e.g., segregation of women in some job positions, with a lower responsibility, higher temporary employment rate and greater instability of employment) that could affect the items regarding the distribution of household tasks and those referring to equality in the work environment are those obtaining the highest score; (b) According to the proposals of Bem (1981) and Markus et al. (1982), sex and gender stereotype will have a direct relationship with maintaining sexist attitudes, so that masculine men and feminine women will have higher scores or, independently from sex, the masculinity and femininity scale will correlate positively with sexism measures. On the other hand, according to Spence’s (1985) proposal no direct relationship would be expected between these variables; (c) Finally, according to the Gender Belief System a significant correlation would be expected between the measures of stereotypes and sexist attitudes.

## Materials and Methods

### Participants and Procedure

Participants included 700 undergraduate students (176 men, 524 women), who ranged in age from 20 to 54 years (*M* = 21.4; *SD* = 4.9). They were enrolled in the third year of psychology graduate studies. Only students who were originally from Spain and reported being heterosexual were selected for this study.

The Ethics Committee of the Autonomous University of Madrid approved this study. Following their statement, all the data for this study was solicited to undergraduate students enrolled in Psychology degree within three different academic terms (2012–2014): all students could choose participation in this study among other equivalently activities; participants completed the instruments in collective sessions in the classrooms; informed consent for participation was previous obtained; anonymity of all participants was assured.

### Measures

#### Bem Sex Role Inventory-12 (Bem, 1974, 1981; Spanish 12-Item Version by Mateo and Fernández, 1991)

It measures self-perceived possession of expressive and instrumental attributes, considered socially desirable for women and men, respectively. The response format is based on a Likert scale from 1 (*never or almost never*) to 7 (*always or almost always*). The short version comprises 12 attributes, six of which represent the dimension of “masculinity” (M) (e.g., “a natural leader”) and other six of “femininity” (F) (e.g., “affective”). For the adaptation into Spanish, Mateo and Fernández (1991) performed a process of translation and back-translation of the original scale. In a more recent study, Fernández and Coello (2010) reported the internal consistency of the BSRI-12 finding a Cronbach’s alpha for the “masculinity” scale of 0.73 and for “femininity” of 0.77. Cronbach’s alpha for the Masculinity scale scores in the sample of men of this study was 0.80 and for women 0.80. The Femininity scale scores showed values of 0.79 for men and 0.82 for women.

#### Modern Sexism Scale (Swim et al., 1995)

It was designed to evaluate subtle or hidden beliefs stating support to gender inequality. It comprises eight items (e.g., “Women often miss out on good jobs due to sexual discrimination”) evaluated in this study in a 5-point Likert-type scale (1 = *strongly disagree* to 5 = *strongly agree*). In the English version the order of the response choices is inverse. However, we decided to keep the same direction of the assessment scale as in the rest of instruments used in the study, so that the lowest scores indicated a greater support to traditional attitudes. In the original version the Cronbach alpha ranged from 0.84–0.75 in different studies (Swim et al., 1995; Swim and Cohen, 1997).

#### Old-Fashioned Sexism Scale (Swim et al., 1995)

This instrument evaluates openly sexist attitudes toward women. It comprises five items (e.g., “I would be equally comfortable having a woman as a boss as a man”) evaluated in a 5-point Likert-type scale (1 = *strongly disagree* to 5 = *strongly agree*). As with the above instrument, in our study the low scores indicate a greater support to sexist attitudes. In the original version the Cronbach’s alpha ranged from 0.66–0.65 in various studies (Swim et al., 1995; Swim and Cohen, 1997). Both scales have been translated into Spanish in a study for a Ph.D. dissertation (Rodríguez, unpublished, cited in Rodríguez et al., 2010); however, no psychometric properties have been reported.

#### Social Roles Questionnaire (Baber and Tucker, 2006)

It comprises 13 items related to the expectations on the behavior that men and women must have in society. Each item is evaluated using a 5-point Likert scale (1 = *strongly disagree* to 5 = *strongly agree*). It is structured in two subscales: “GT” with five items (e.g., “People should be treated the same regardless of their sex”) that evaluates the support on the attitudes which keep a non-dichotomous gender view; and “Gender Linked” (GL) with eight items (e.g., “Mothers should work only if necessary”) that evaluates the beliefs about the association of some activities with one or the other gender. The original study reported Cronbach’s alpha values of 0.65 for GT and 0.77 for GL. In our study, we have recoded inversely the items from the “GT” factor, following the indications of the original authors, so that the interpretation of the scores obtained in both factors is performed with the same meaning: high scores indicate a greater support to sexist attitudes. A Spanish version (López-Cepero et al., 2013) was examined through a CFA finding sufficient support to the two-factor solution. However, the internal consistency of the items for the “GT” factor was lower than 0.47 in men and women, and the “Gender Liked” factor was 0.77. These data led the authors to recommend the review of the translation into Spanish for subsequent use.

### Back-Translation of the SRQ, MS, and OFS Spanish Versions

The International Test Commission has established a methodological standard to adequately adapt instruments from one culture to a new one (Hambleton, 1994, 1996; Muñiz et al., 2013). Following these recommendations, the first step was to evaluate the possible influence of the cultural and linguistic differences in the Spanish context. In this regard, a team of five expert investigators provided advice. Later, two qualified translators, one of them of Spanish origin and the other one of English origin, were trained on the constructs evaluated and they translated and back-translated all the items. The expert team help to evaluate the equivalence between the two original version and the Spanish version, making the appropriate changes in the new versions.

### Data Analyses

The software SPSS version 19.0 (IBM Corp, 2010) and EQS 6.1 (Multivariate Software, Inc., Bentler, 1995) were used for data analyses. The scores for each item were compared between the samples of men and women using the student’s *t*-test. An analysis of the effect power was also performed using the Cohen’s (1988) *d* statistics to estimate the magnitude of the result obtained (Wilkinson et al., 1999). A value of 0.2 would correspond to a small effect size, 0.5 moderate and 0.8 high (Cohen, 1988).

Confirmatory factor analysis were conducted on the SRQ items for the men and women groups. Three different models were tested: (1) the original two-factor model (Baber and Tucker, 2006), and (2) a one-factor model that included all of the items of the SRQ. For the MS and OFS scales the following models were examined for men and women: (1) original one-factor models for both scales (Swim et al., 1995; Swim and Cohen, 1997), and (2) a joint model with two related factors corresponding to each scale. In all cases, the model fit was evaluated considering the following fit indices: the comparative fit index (CFI; Bentler, 1990), the non-normed fit index (NNFI; Bentler and Bonett, 1980), the root mean square error of approximation (RMSEA; Steiger, 1990), and the standardized root mean square residual (SRMR; Jöreskog and Sörbom, 1996). CFI and NNFI values greater than 0.90, RMSEA less than 0.06 and SMRS less than 0.08 (Byrne, 1994; Cheung and Rensvold, 2002) evidence a good model fit.

Multigroup CFA models were conducted to examine the invariance across the men and women samples of the components of the model and the underlying theoretical structure (Byrne et al., 1989). In order to assess for measurement invariance across the samples, it was used the robust maximum likelihood estimation. Testing for equivalence based on the analysis of means and covariance structures follows a set of steps (Byrne, 2008): (1) first, to determinate a good multigroup baseline model fit, (2) Model 1: a configural model is the least restrictive model; it consist of testing the same configuration of fixed and freely estimated parameters with no equality constraints, (3) Model 2: it implies the constraints of observed variables and their links to the latent variables (i.e., factor loadings), (4) Model 3: it involves the unobserved variables too (i.e., factor covariances), (5) Model 4: it adds to the previous ones the intercepts invariant, and finally (6) testing for latent means differences between groups. We used two indicators to test invariance: (1) the corrected scale S–B χ^2^ difference test developed by Satorra and Bentler (2001). If this difference is statistically significant, it indicates that the constraints specified in the model do not hold; (2) the changes in CFI, as a less vulnerable indicator to variations in sample size and non-normality (Cheung and Rensvold, 2002; Cheung, 2008). This difference should not exceed 0.01.

To analyse the empirical relationship between SRQ, MS, and OFS measures and the BSRI instrument, the following analyses were performed: (1) bivariate correlations between all scales, dividing the sample into men and women; and (2) classify the sample according to the score obtained in the “Masculinity” (M) and “Femininity” (F) scales of the BSRI in four groups (“Undifferentiated,” “Masculine,” “Feminine” and “Androgynous”), taking the median as a cut-off point (for men in the M scale it was 4.7 and in the F scale it was 5.5; for women 4.4 and 5.7 for M and F, respectively); finally, given the non-normal group distribution by sex and the classification of the BSRI, as well as the different groups sizes, Kruskal-Wallis tests were performed to analyse the differences in the score ranges obtained in SRQ, MS, and OFS between the different groups.

## Results

### Reliability: Internal Consistency

The Cronbach’s alpha coefficients attained low but sufficient values for the subscales scores of SQR: males “GT” (α = 0.6) and “GL” (0.8); Females “GT” (0.6) and “GL” (0.7). Appropriate Cronbach’s alpha values were found for the MS in the group of men (0.8) and women (0.8); however, low values were found for the OFS scores in both the group of men (0.5) and of women (0.5).

### Item Analysis

The means, standard deviations and item-total correlations of the SRQ items are shown in Table 1. Several items showed an item-total correlation value under 0.3: in the case of men item 1 and in the case of women also item 1, in addition to item 6 and 10. In respect to the mean scores obtained by men and women in the global scores of the subscales, significant differences were found with a moderate effect size in GL and small in GT, in both cases men having higher scores. This was also the case for items showing significant differences, with small effect sizes for all those of the GT subscale, and most of the GL items, but for item 8 (moderate).

**TABLE 1:** Descriptive statistics and factor loadings (CFA two-factor model) and mean scores comparison of SRQ for males (*n* = 176) and females (*n* = 524).

	**Males**			**Females**					
**Items**	***M* (*DT*)**	***r*^c^_iX_**	**Factor loadings**	***M* (*DT*)**	***r*^c^_iX_**	**Factor loadings**	**Student *t***	**df**	**Cohen’s *d***
GT	1.4 (0.5)			1.3 (0.4)			2.586^∗∗^	248,384	0.27
Item 1	1.4 (0.8)	0.25	0.382	1.4 (0.8)	0.24	0.305	0.121	698	0.01
Item 2	1.4 (0.8)	0.34	0.427	1.2 (0.5)	0.44	0.487	2.755^∗∗^	212,756	0.31
Item 3	1.3 (0.7)	0.49	0.646	1.3 (0.6)	0.47	0.708	1,237	273,940	0.12
Item 4	1.2 (0.5)	0.52	0.701	1.1 (0.4)	0.31	0.416	2.818^∗∗^	234,930	0.29
Item 5	1.5 (0.8)	0.47	0.544	1.4 (0.7)	0.41	0.596	2.046^∗^	260,525	0.20
GL	1.8 (0.6)			1.6 (0.5)			5.144^∗∗^	248,174	0.51
Item 6	2.2 (1.1)	0.33	0.355	1.9 (0.9)	0.29	0.300	3.200^∗∗^	267,372	0.30
Item 7	2.3 (1.2)	0.40	0.433	2.1 (1.1)	0.39	0.452	1,730	279,735	0.16
Item 8	2.4 (1.3)	0.51	0.621	1.8 (1.0)	0.56	0.722	5.125^∗∗^	248,845	0.50
Item 9	1.5 (0.7)	0.34	0.399	1.4 (0.7)	0.46	0.479	0.193	698	0.01
Item 10	1.3 (0.7)	0.42	0.536	1.1 (0.4)	0.29	0.317	3.298^∗∗^	225,041	0.35
Item 11	1.9 (1.1)	0.50	0.578	1.6 (0.9)	0.36	0.469	2.992^∗∗^	260,921	0.28
Item 12	1.8 (1.1)	0.58	0.719	1.4 (0.8)	0.51	0.687	3.796^∗∗^	245,542	0.38
Item 13	1.4 (0.8)	0.53	0.592	1.2 (0.5)	0.30	0.370	3.994^∗∗^	223,873	0.43

*^∗∗^*p* < 0.01; ^∗^*p* < 0.05. *r*^*c*^_*iX*_: item-total correlation. GT = Gender Transcendent; GL = Gender Linked.*

The same descriptive data for the MS and OFS instruments are shown in Table 2. The MS items evidenced item-total correlations above 0.4, except for item 8. The OFS instrument items obtained item-total correlations values between 0.2 and 0.3 in men and between 0.2 and 0.3 in women. The differences in the mean global scores of both instruments between men and women were significant and with a moderate effect size in both cases. Women’s scores were higher in all items of both scales, indicating a lower support to traditional sexist attitudes. Effect sizes were generally small for MS (but for item 7, moderate) and OFS items.

**TABLE 2:** Descriptive statistics and factor loadings (CFA two-factor merge model) and mean scores comparison of MS and OFS for males (*n* = 176) and females (*n* = 524).

	**Males**			**Females**					
**Items**	***M* (*DT*)**	***r*^c^_iX_**	**Factor loadings**	***M* (*DT*)**	***r*^c^_iX_**	**Factor loadings**	**Student *t***	**df**	**Cohen’s *d***
MS	3.7 (0.6)			3.9 (0.6)			−4.173^∗∗^	696	0.37
Item 1	4.0 (0.9)	0.64	0.816	4.2 (0.8)	0.57	0.659	−2.762^∗∗^	695	0.26
Item 2	3.9 (0.9)	0.44	0.510	4.1 (0.9)	0.39	0.455	−0.809	696	0.08
Item 3	4.1 (1)	0.45	0.551	4.1 (1)	0.46	0.523	−0.809	696	0.07
Item 4	4.1 (0.9)	0.49	0.610	4.1 (0.9)	0.57	0.639	0.033	696	0.00
Item 5	3.5 (1)	0.49	0.608	3.7 (0.9)	0.53	0.604	−2.182^∗^	696	0.19
Item 6	3.3 (1.1)	0.45	0.458	3.7 (0.9)	0.52	0.612	−4.923^∗∗^	271,750	0.45
Item 7	3.6 (1.1)	0.49	0.493	4.1 (0.8)	0.58	0.669	−5.311^∗∗^	246,721	0.53
Item 8	3.2 (1.1)	0.31	0.324	3.5 (1.1)	0.35	0.398	−3.062^∗∗^	696	0.27
OFS	4.6 (0.5)			4.7 (0.4)			−3.685^∗∗^	257,682	0.35
Item 1	4.7 (0.8)	0.24	0.308	4.8 (0.7)	0.20	0.279	−1.948^∗^	261,137	0.18
Item 2	4.6 (0.8)	0.31	0.414	4.7 (0.7)	0.16	0.261	−2.247^∗^	260,563	0.21
Item 3	4.7 (0.9)	0.29	0.493	4.8 (0.7)	0.32	0.500	−1,275	261,452	0.12
Item 4	4.5 (1)	0.26	0.373	4.7 (0.9)	0.20	0.282	−2.256^∗^	276,778	0.21
Item 5	4.5 (0.8)	0.24	0.407	4.7 (0.6)	0.33	0.610	−2.839^∗∗^	243,967	0.29

*^∗∗^*p* < 0.01; ^∗^*p* < 0.05. *r*^*c*^_*iX*_: item-total correlation. MS = Modern Sexism; GL = Old-Fashioned Sexism.*

### Factor Analyses

The fit indices for the factorial analyses of the SRQ models are shown in Table 3. The two-factor model reached appropriate levels of fit in male and female samples and yielded higher values of CFI and NNFI and a lower S–B χ^2^ value than the one-factor model.

**TABLE 3:** Summary of fit indices for the CFAs of SRQ for males (*n* = 176) and females (*n* = 524).

**Model**	**S**–**B χ^2^**	**CFI**	**NNFI**	**SRMR**	**RMSEA**
**Males**					
One-factor model	115,128	0.827	0.793	0.074	0.066 (CI:0.046, 0.086)
Two-factor model	90,258	0.909	0.890	0.064	0.048 (CI:0.021, 0.070)
**Females**					
One-factor model	184,024	0.806	0.768	0.060	0.059 (CI:0.049, 0.069)
Two-factor model	112,055	0.922	0.905	0.046	0.034 (CI:0.026, 0.049)

*S–B χ^2^ = Satorra–Bentler scaled chi-square; CFI = comparative fit index; NNFI = non-normed fit index; RMSEA = root mean square error of approximation; SRMR = standardized root mean square residual; CI = 90% confidence interval.*

For the CFA of the MS and OFS scales, a double approach was used (Table 4): first, the one-factor models were examined for each scale in both sexes, finding poorly appropriate values for the fit (low CFI and NNFI and high RMSEA values) in the group of men. In the case of women the one-factor model for MS did not obtain appropriate values either, but did for the OFS scale (CFI and NNFI close to 1 and low RMSEA). Second, given the disparity found in both sexes and considering the high correlation between the scales, the fit of a two-factor model with the items of each scale grouped as correlated factors was examined (two-factor merge model). This model found sufficiently appropriate values in both sexes, so the factorial invariance analysis was continued. Factor loadings of the MS and OFS items on the two-factor merge model are shown in Table 2.

**TABLE 4:** Summary of fit indices for the CFAs of MS and OFS for males (*n* = 176) and females (*n* = 524).

**Model**	**S**–**B χ^2^**	**CFI**	**NNFI**	**SRMR**	**RMSEA**
**Males**					
One-factor MS model	51,903	0.860	0.804	0.072	0.096 (CI:0.064, 0.128)
One-factor OFS model	9,786	0.813	0.627	0.048	0.074 (CI:0.000, 0.142)
Two-factor merge model	95,440	0.891	0.867	0.067	0.053 (CI:0.029, 0.074)
**Females**					
One-factor MS model	134,646	0.864	0.810	0.058	0.105 (CI:0.088, 0.122)
One-factor OFS model	5,930	0.978	0.956	0.029	0.019 (CI:0.000, 0.066)
Two-factor merge model	194,149	0.854	0.823	0.054	0.062 (CI:0.052, 0.072)

*S–B χ^2^ = Satorra–Bentler scaled chi-square; CFI = comparative fit index; NNFI = non-normed fit index; RMSEA = root mean square error of approximation; SRMR = standardized root mean square residual; CI = 90% confidence interval.*

### Measurement Invariance

For the SRQ, the configural model (Model 1) indicated that the multigroup model fit well across the male and female samples (Table 5). The comparison of Model 2 and configural model was shown to be non-invariant, so the modification indices offered by the LM test were followed, suggesting release of non-invariant items 4 (*p* = 0.00) and 13 (*p* = 0.01). Once released, the modified Model 2 was compared with the configural model, indicators evidencing equivalence. For the following models both items were kept released, so that the assessment of the partial invariance of the instrument was continued. Equivalence Model 3 was adequate, the ΔS–B χ^2^ was not significant and the ΔCFI was <0.01, reflecting partial invariance compared to the configural model. In the comparison of Model 4 and the configural model, ΔS–B χ^2^ was significant, despite ΔCFI being <0.01. The results of the LM test suggested modifications for several intercept constraints. So, we may conclude that the intercept equivalence was not achieved, and we didn’t further continue to compare the latent means.

**TABLE 5:** Measurement invariance of SRQ (two-factor model) for males (*n* = 176) and females (*n* = 524).

	**S**–**B χ^2^**	**CFI**	**NNFI**	**SRMR**	**RMSEA**	**ΔS**–**B χ^2^**	**ΔCFI**
Model 1 Configural	202,922	0.919	0.901	0.056	0.029 (CI:0.021, 0.036)		
Model 2 Factor loadings	322,536	0.896	0.917	0.083	0.031 (CI:0.024, 0.038)	29.026 (df = 11, *p* = 0.00)	0.023
Model 2 modified Items 4 and 13 free	294,275	0.913	0.901	0.041	0.029 (CI:0.021, 0.036)	14.442 (df = 9, *p* = 0.12)	0.006
Model 3 Covariances	298,179	0.912	0.900	0.078	0.029 (CI:0.021, 0.036)	19.081 (df = 10, *p* = 0.10)	0.007
Model 4 Intercepts	341,824	0.912	0.895	0.076	0.033 (CI:0.026, 0.039)	57.778 (df = 19; *p* = 0.00)	0.007

*S–B χ^2^ = Satorra–Bentler scaled chi-square; CFI = comparative fit index; NNFI = non-normed fit index; RMSEA = root mean square error of approximation; SRMR = standardized root mean square residual; CI = 90% confidence interval.*

The factorial invariance analysis of the two-factor model of MS and OFS (Table 6) found adequate values of the equivalence of the factor loadings and covariances (Model 3), but Model 4, as in the case of SRQ, indicated the lack of equivalence of the intercepts: ΔS–B χ^2^ was significant, despite ΔCFI being <0.01. So, we may conclude that the intercept equivalence was not achieved.

**TABLE 6:** Measurement invariance of MS and OFS (two-factor merge model) for males (*n* = 176) and females (*n* = 524).

	**S**–**B χ^2^**	**CFI**	**NNFI**	**SRMR**	**RMSEA**	**ΔS**–**B χ^2^**	**ΔCFI**
Model 1 Configural	297,509	0.861	0.830	0.061	0.044 (CI:0.037, 0.050)		
Model 2 Factor loadings	307,743	0.861	0.844	0.065	0.042 (CI:0.024, 0.038)	11.013 (df = 11, *p* = 0.44)	0.000
Model 3 Covariances	308,822	0.861	0.845	0.065	0.042 (CI:0.035, 0.048)	11.601 (df = 12, *p* = 0.48)	0.000
Model 4 Intercepts	372,798	0.865	0.839	0.068	0.046 (CI:0.040, 0.052)	79.523 (df = 23; *p* = 0.00)	0.004

*S–B χ^2^ = Satorra–Bentler scaled chi-square; CFI = comparative fit index; NNFI = non-normed fit index; RMSEA = root mean square error of approximation; SRMR = standardized root mean square residual; CI = 90% confidence interval.*

### Bivariate Correlations

The subscales of the SRQ instrument correlated to each other significantly in both sexes, as with the MF and OFS instruments with each other also in men and women (Table 7). The SRQ subscales had significant negative relationships with MS and OFS in both sexes, except for the “GL” subscale and the MS instrument in the group of men, which was non-significant. In the case of the BSRI, only a significant relationship was found: both in men and women, the “GT” subscale was positively related to the male stereotype in the case of men and negative in women. Correlations were small for women and small to moderate for men.

**TABLE 7:** Correlations for SRQ, MS, OFS, and BSRI for males (*n* = 176) and females (*n* = 524).

	**SRQ**	**SRQ**	**OFS**	**MS**	**BSRI**	**BSRI**
	**– GT**	**– GL**			**– MASC**	**– FEM**
SRQ – GT	1	0.47^∗∗^	–0.32^∗∗^	–0.26^∗∗^	0.17^∗^	–0.15
SRQ – GL	0.40^∗∗^	1	−32^∗∗^	0.12	0.12	–0.11
OFS	–0.13^∗∗^	–0.13^∗∗^	1	0.16^∗^	–0.02	0.09
MS	−0.09^∗^	–0.23^∗∗^	0.17^∗∗^	1	–0.14	0.03
BSRI – MASC	−0.10^∗^	–0.02	0.02	–0.03	1	0.04
BSRI – FEM	–0.05	–0.08	–0.02	–0.05	0.04	1

*^∗∗^*p* < 0.01; ^∗^*p* < 0.05. SRQ – GT = Social Role Questionnaire Gender Transcendent; SRQ – GL = Social Role Questionnaire Gender Linked; OFS = Old-Fashioned Sexism; MS = Modern Sexism; BSRI – MASC = Bem Sexual Role Inventory Masculine; BSRI – FEM = Bem Sexual Role Inventory Feminine. The correlations of sample of males are shown in the upper right corner and the sample of females in the lower left corner.*

### Mean Comparison Across Gender Stereotypes Classification

First, the median-split method was used for the classification of the sample in the four stereotypes measured by the BSRI. Table 8 shows the distribution by stereotype for men and women. A chi-square test was performed, finding no significant differences in the sample distribution by sex and gender stereotypes (χ^2^ = 1.62; *p* = 0.66).

**TABLE 8:** Distribution of males (*n* = 176) and females (*n* = 524) across gender stereotypes classification.

	**Undifferentiated**	**Masculine**	**Feminine**	**Androgynous**
	***N* (%)**	***N* (%)**	***N* (%)**	***N* (%)**
Males	63 (35.8)	39 (22.2)	34 (19.3)	40 (22.7)
Females	197 (37.6)	112 (21.4)	116 (22.1)	99 (18.9)
Total	260 (37.1)	151 (21.6)	150 (21.4)	139 (19.9)

*Percentages are over the total number of men and women.*

Second, several Kruskal-Wallis tests were performed to compare the ranks for the instruments between the groups distributed by sex and gender stereotypes, finding significant differences in the scores of the “GL” subscale of SRQ (*p* = 0.00) and of the OFS (0.00) and MS instruments (0.02). Table 9 shows the means and the standard deviations. The significant relationships are shown below for pair comparisons:

**TABLE 9:** Descriptive statistics of males (*n* = 176) and females (*n* = 524) across gender stereotypes classification.

	**Males**				**Females**			
	**Indiferenciate**	**Masculine**	**Feminine**	**Androgynous**	**Indiferenciate**	**Masculine**	**Feminine**	**Androgynous**
	***M* (*SD*)**	***M* (*SD*)**	***M* (*SD*)**	***M* (*SD*)**	***M* (*SD*)**	***M* (*SD*)**	***M* (*SD*)**	***M* (*SD*)**
SRQ – GT	1.4 (0.5)	1.5 (0.5)	1.3 (0.3)	1.4 (0.5)	1.3 (0.4)	1.2 (0.4)	1.2 (0.3)	1.3 (0.3)
SRQ – GL	1.9 (0.6)	2 (0.6)	1.7 (0.6)	1.8 (0.6)	1.6 (0.5)	1.6 (0.5)	1.5 (0.5)	1.6 (0.5)
OFS	4.5 (0.5)	4.6 (0.6)	4.7 (0.5)	4.6 (0.5)	4.7 (0.4)	4.8 (0.3)	4.8 (0.3)	4.7 (0.5)
MS	3.8 (0.6)	3.6 (0.7)	3.8 (0.5)	3.6 (0.7)	3.9 (0.6)	3.9 (0.6)	3.9 (0.6)	3.9 (0.6)

*SRQ – GT = Social Role Questionnaire Gender Transcendent; SRQ – GL = Social Role Questionnaire Gender Linked; OFS = Old-Fashioned Sexism; MS = Modern Sexism.*

• SRQ “GL” subscale: significant differences were found in the comparison of the group of “masculine” males and all groups of women: “undifferentiated” (*p* = 0.01), “masculine” (0.01), “feminine” (0.00), and “androgynous” (0.00). Significant differences were found between the group of “undifferentiated” men and that of “feminine” women (0.00).
• OFS: significant differences were found between the group of “undifferentiated” men and several groups of women: “undifferentiated” (*p* = 0.00), “masculine” (0.00), and “feminine” (0.00).

## Discussion

### On the Psychometric Properties of Instruments

The results of this study provided additional evidence of the reliability, validity, and cross-cultural adequacy of the SRQ, OFS, and MS scales. With regard to the factorial structure of the instruments, as expected, fit indicators were obtained that supported the original two-factor structure of the Spanish version of the SRQ in both sexes (Baber and Tucker, 2006). However, in the case of the MS and OFS scales, the MS alone model exhibited a less than ideal fit to the data, but the OFS alone model showed a good fit to the observed data in the group of women. Given the high correlation between the scales in both sexes, the fit of a two-factor model was examined with the set of items of both scales (two-factor merge model), exhibiting adequate fit indicators, in line with the proposal of the original authors (Swim et al., 1995; Swim and Cohen, 1997).

With regard to the internal consistency of the Spanish versions of the instruments, values were comparable to those obtained in previous studies. The GT subscale of the SRQ exhibited moderate internal consistency values in both sexes, superior, however, to those found by López-Cepero et al. (2013). Less than ideal values consistently found for GT in Spanish samples might be related to the reduced number of items and all items being inversely worded. To be noted is the scarce relation of item 1 (“*Persons can be both aggressive and affectionate, regardless of their sex*”) to the rest of the items of GT for men and women. It is the only item making reference to a violent behavior, maybe relating to a very specific issue, which is the justification of violence in some contexts and its relationship with the gender differences in partner relations (Corral and Calvete, 2006; Garaigordobil et al., 2013). This might be markedly far from the content of the rest of the scale items. Of the other two scales, the Spanish version of the OFS exhibited questionable poor values in both sexes, lower to those moderate values reported elsewhere (Swim et al., 1995; Swim and Cohen, 1997). Again, there is a potential detrimental effect of the shortness of the scale, as well as the fact that three of its five items are inverse. Item 2 (“*I would be equally comfortable having a woman as a boss as a man*”) showed the lowest relation with the rest of items in the group of women. The content of item 2, alludes to a prejudice that might has not been overcome in the same consistent way as the rest of old-fashioned attitudes included in the instrument (e.g., recognizing that men and women have the same intellectual capacity). With regard to this, several studies have found that female leaders usually receive worse satisfaction ratings from their subordinates in some organizational contexts (Eagly et al., 1995; Cuadrado, 2003).

### Differences Between Men and Women in the Assessment of Sexist Attitudes

The comparison among the scores exhibited by men and women in the items of the Spanish versions of the SRQ, OFS, and MS scales, and the assessment of invariance, provided interesting results. In all cases men expressed a greater support to sexist attitudes, which is consistent with previous studies using the same instruments or similar (Swim et al., 1995; Glick and Fiske, 1996; Campbell et al., 1997; Swim and Cohen, 1997; Baber and Tucker, 2006). For the SRQ, Item 8 (“*Some jobs are not appropriate for women*”) showed the greater difference among men and women. Its content may gather one of the sexist attitudes currently most established in the Spanish society where in fact we find very remarkable differences in the presence of men and women in some work areas (Heilman and Eagly, 2008; Bonilla, 2010; Gino et al., 2015), that grow in all cases in the power positions of the organizational hierarchy. According to the last reports of the Spain’s Institute for Women, as indicated previously, women hold the job positions which are most temporal, part-time, with worst conditions and fewer employment rights. To be noted is the fact that men expressed a greater support to the sexist attitudes shown in the SRQ for considering gender as an essential factor when distributing tasks both at home and at work. These data are consistent with those found in other studies where men also expressed more overt sexist attitudes than women (Moya et al., 2007; Sibley et al., 2007). With regard to the differences in the analysis of the scores obtained by men and women in the MS and OFS items, as stated, women showed more egalitarian attitudes, contrary to sexism, evidenced in this case by higher scores, especially for Item 7 (“*It is easy to understand why feminist groups are still concerned about the social limitations in the opportunities for women*”). We find coherent that women obtain higher scores, as it has been shown that they adhere to a feminist identity to a higher degree (Zucker and Bay-Cheng, 2010; Parry, 2014), they understand better the claims of feminist groups and give their support (Hooks, 2000). In the case of OFS, though a small size effect was found, outstand Item 5 (“*When both parents work and their child gets sick, the school should call the mother rather than the father*”), which content is related to assigning household and care tasks in the family environment, finding in this a very marked sexist trend in Spain and in the rest of the Western countries (Instituto de la Mujer, 2016; United States Bureau of Labor Statistics, 2016).

In this study, we have tested the equivalence across sex of the factor structure, factor loadings and intercepts. The factor structure invariance (Model 1) and loadings (Model 2 and 3) was supported by SRQ, MS, and OFS. The joint model for MS and OFS obtained adequate values of goodness of fit at this comparison level; however, for SRQ Model 2 indicated the lack of equivalence suggesting the release of two items that were invariant between sexes (4: “*Household chores should not be assigned by sexes*”; 13: “*In many important jobs it is better to contract men than women*”). Once both items were released (Model 2 modified) adequate equivalence values were obtained both in this model and in Model 3. Both items exhibited significant differences in the mean scores, with men attaining higher scores. The content of the items mention two highly significant subjects related to the evolution of gender roles in Spain, that we have already discussed; on the one hand, household chores are still distributed very unequally in our society, and women fulfill these tasks almost alone as a general rule, despite their joining the labor market (Silván-Ferrero and Bustillos, 2007); and on the other hand the unequal rating received by male and female leaders in senior posts of the organizational hierarchy (Eagly et al., 1995; Cuadrado, 2003). In conclusion, these are two sexist prejudices that have not been yet overcome *de facto* in our society, which could explain the different performance of these items when asking men and women.

At last, Model 4 tests whether an item has the same point of origin across different groups. For the Spanish versions of the instruments, we may conclude that intercept invariance is not achieved in either case, so the scores from men and women have not the same origin. Several factors can affect the origin of a scale, as pointed by Chen (2008): social desirability; the trend to show a strong desire for values involving a defect or deficit for the group to which one belongs; the cultural reference framework to which one belongs and from which self-judgments are made. In respect to these statements, in our study we have not included any measure of social desirability, so we cannot establish differences between men and women with this regard. However, differences shown in this level of measurement invariance, may lead to the questions: Does the Spanish cultural framework justify gender differences in our society? Have sufficient political or social achievements been obtained to overcome sexist attitudes? Are overt and subtle sexist attitudes held in the same way by men and women in our society? To discuss these questions we consider relevant to briefly discuss some sociological data that can help us understand the sociocultural framework of Spain with regard to gender differences. With this regard, several studies have noted that in the Spanish context sexist attitudes that denigrate and place women in a clear position of inequality are justified and held. One of the most clear examples is the unequal access to the labor market, and today there are disciplines fully polarized in terms of distribution by sex (in the Building sector only 7.6% are contracted and in the Industry 25.1%; Instituto de la Mujer, 2016), the inequality in salaries (women with permanent contracts are paid 25.7% less than men and in temporary contracts 10.4% less; Instituto de la Mujer, 2016) or the lack of opportunities for rising to power positions in the organizational hierarchy (in the level of “Managers and Directors” we find 31.4% women and 20.7% in governing boards of the companies of Ibex-35; Instituto de la Mujer, 2016). All of this leads us to the conclusion that in our country a great effort is still needed to overcome the lack of opportunity, rights and freedoms assigned to persons on the basis of sex (García-Dauder, 2005).

### Are We Assessing the Same Issue With Different Instruments?

The correlations obtained between the scores of the three instruments were those expected, as the interpretation of the MS and OF scores on the one hand and the SRQ on the other, is the opposite, that is, the higher the MF and OFS scores, the lower the support to sexist attitudes, and the higher the scores in the two SRQ scores, the greater the support to sexist attitudes.

However, given the lack of invariance in the three instruments in terms of intercepts (Model 4), we question if the existing instruments perform a sufficient, comprehensive approach to understand and explain the persistence of sexist attitudes in men and women in today’s society. On the one hand, the validity of the instruments used to measure gender stereotypes and attitudes on gender roles must be analyzed critically. In none of these instruments the evaluation of personal beliefs, understanding of cultural stereotypes, sexist prejudices or degree of consistency between behavior and said stereotypes is distinguished (Zosuls et al., 2011). Those are clearly different contents on which the necessary effort has not been made to distinguish the multidimensional composition of the gender stereotypes and their characterization with the appropriate sensitivity to the differences between men and women.

On the other hand, the need for performing current qualitative studies gathering the social representation of the clearest inequalities in society should be also discussed. Although recent efforts have been made in the design of new self-reports (Baber and Tucker, 2006; García-Cueto et al., 2015), their proposal is clearly continuist in terms of the content and wording of the items. Future instrumental studies might benefit from considering different dimensions gathering various thematic areas where sexist attitudes are shown, such as the work environment, at home and within interpersonal relations, as well as traits and personal skills attributed to each sex. In this respect, a notable progress has been made by our society in terms of overcoming some of these attitudes as a result of social and political achievements of recent years (e.g., Laws on Gender Equality, 2007, and Same Sex Marriage, 2005), but there are other areas in which attitudes persist that justify serious differences between men and women, limiting the latter to an unfair inferior position.

### Are Gender Stereotypes and Gender Roles Attitudes Linked?

In our study we have related gender stereotypes and gender roles attitudes in two different ways: (1) analyzing the correlations between the BSRI scales and instruments evaluating sexist attitudes; and (2) classifying the sample according to the prevailing stereotype and comparing scores in the instruments measuring sexism among the different groups. For the first approach, contrary to the expectations based on the Gender Schema Theories (Bem, 1981; Markus et al., 1982) and Gender Belief System (Deaux and Kite, 1987; Kite, 2001), in the line of a direct relationship between holding traditional stereotypes and justifying gender differences, we found that the only significant correlations were between the subscale of “masculinity” and the GT subscale of SRQ, positive for men and negative for women.

## Conclusion

We provided additional evidence that instruments have not been updated or reformulated according to the social changes occurring in the past decades in terms of the position of women in society and the evolution of sexist attitudes in the Western culture framework (Twenge, 1997; Hoffman and Borders, 2001). With this regard, we recommend a critical analysis of the psychometric properties of instruments of over three decades of age as urgent and necessary, as well as an update of their theoretical foundations and reformulations in order to reflect both the social roles and the stereotypes representative of the images of men and women in today’s society.

This work shares common limitations, mainly related to the extraction and characteristics of the sample composed of relatively homogeneous groups of undergraduate students. In order to examine the psychometric properties of the instruments, we thought it preferable to exclude those participants with diverse original language and/or cultural backgrounds and sexual orientations, other than Spanish and heterosexual, respectively. The numbers of participants who were originally from other countries, whether Spanish-speaking or not, and non-heterosexual, were relatively small as to make it possible to do separate analyses. Thus, the rationale has to do with trying to ensure appropriate understating of the items and address possible nuances related to diversity. In this regard, future research should study heterogeneous samples in terms of sexual and gender diversity and would benefit from using qualitative methodologies to collect personal experiences in a comprehensive way.

Future investigations should also collect larger, diverse samples in terms of age, educational level, and occupation and, in this way, check that findings can be extrapolated to the rest of the Spanish population. Finally, in the light of our data and being aware of the limitations of our study (in terms of origin of the sample, exclusive use of self-report measures and their age), the results of this study questioned the validity and effectiveness of the classic theories in Gender Psychology, like Gender Schema Theories (Bem, 1981; Markus et al., 1982) and Gender Belief System (Deaux and Kite, 1987; Kite, 2001), in terms of the lack of empirical support to the expected link between the sexist attitudes and the traditional gender stereotypes. As noted in the first place by Spence (1985), it might be suggested that the consistency between role, stereotype and identity shouldn’t necessarily be expected, according to the last formulations on the fluidity of gender in the queer theory (Butler, 1990, 2004; Preciado, 2008) and transgender theory (Nagoshi et al., 2014). With this regard, the construction of gender is in constant evolution and such transformation is transversal to the components thereof but needs not affect all in the same way. Therefore, we could introduce alternative esthetic elements according to our gender and still maintain homophobic or male chauvinist attitudes. The influence of the social and political landmarks reached and the openness in terms of socially permitted manifestations, attitudes or behaviors are the grounds of a major process of change in Western societies (Nagoshi et al., 2012). In conclusion, in such a changing situation in terms of the sociocultural construction of gender, it is necessary to challenge the predictive capacity of static and old models or theories not acknowledging social changes or the fluidity of gender in our context.

## Data Availability Statement

All datasets generated for this study are included in the article/supplementary material.

## Ethics Statement

The studies involving human participants were reviewed and approved by the Ethics Committee of the Autonomous University of Madrid. The patients/participants provided their written informed consent to participate in this study.

## Author Contributions

RG-S and CA carried out the initial theoretical revision, the data analysis, and elaborated the discussion section. BA, MM, and MS-O were responsible for collection of the data and participated in the data analysis. JL and JM participated in the writing of the discussion and in the elaboration of the overall conclusion.

## Conflict of Interest

The authors declare that the research was conducted in the absence of any commercial or financial relationships that could be construed as a potential conflict of interest.
